# Soil-transmitted helminthiasis and undernutrition among schoolchildren in Mettu town, Southwest Ethiopia

**DOI:** 10.1038/s41598-022-07669-4

**Published:** 2022-03-07

**Authors:** Solomon Yeshanew, Teshome Bekana, Zemenay Truneh, Melaku Tadege, Embiet Abich, Habtamu Dessie

**Affiliations:** 1 Department of Biology, Debre Markos University, Debre Markos, Ethiopia; 2 Department of Biomedical Sciences, Mettu University, Mettu, Ethiopia; 3 Department of Midwifery, Debre Markos University, Debre Markos, Ethiopia; 4 Department of Statistics, Injibara University, Injibara, Ethiopia

**Keywords:** Nutrition, Paediatrics, Public health, Health care, Health occupations, Medical research

## Abstract

Soil-transmitted helminthiasis (STHs) and undernutrition are common health problems in developing countries. Several reports showed that STH and undernutrition are often associated. The main aim of this study was to determine the association of STH and undernutrition among schoolchildren in Mettu town, Southwest Ethiopia. A cross-sectional study design was employed. To collect socio-demographic data, semi-structured questionnaire and physical observation were used. Kato-Katz technique and Anthropometric measurements were also considered to see STH infection and determine the nutritional statuses of the study participants respectively. Then, the data generated from the study was managed using Chi-square test and logistic regression analysis to determine the association of demographic variables with infections of helminthes and assess the risk factors for nutritional status of the study participants respectively. As a result, among the 392 study schoolchildren, 331 (84.4%) children were positive for different species of STH and undernutrition accounted 32.6%. *Ascaris lumbricoides* (39.0%), *Trichuris trichiura* (32.9%) and hookworm (28.1%) are the predominant STH identified from the study participants. Age, maternal educational and occupation status, and fingernail status of children were found significantly associated (p < 0.05) with the risk of getting STH. Bivariate logistic regression analysis showed that, age (AOR 2.18, 95% CI 1.53, 6.59), maternal illiteracy (AOR 0.13, 95% CI 0.91, 0.34) and maternal occupation (AOR 1.67, 95% CI 1.08, 5.91) were major co-founding factors for the prevalence of STH among study participants. In addition, children with *T. trichiura* infection were more likely (P < 0.01) to suffer from undernutrition (AOR 0.52, 95% CI 0.31, 0.83). Thus, the findings revealed the high prevalence of STH and it has significant association with undernutrition among school age children in the study area. Anti-helminthic mass drug administration and maternal health education should be anticipated to curve the tragedy.

## Introduction

STH is among the most common infectious diseases worldwide and affects the poorest and most deprived communities. *A. lumbricoides*, *T. trichiura,* and Hookworm (*Necatoramericanus*and *Ancylostomaduodenale)* are the three major STH that are prevalent in developing countries and characterized by having low socioeconomic status such as poor housing and sanitation conditions, unsafe water supplies, inefficient or no health care, poor education, and low remunerations^1,2^.

STH infections have been found in several studies to be associated with malnutrition and anemia^3,4^. Malnutrition and STH infections often coexist in the same geographical locations with the same individuals experiencing both conditions. Protein-energy malnutrition (PEM) and iron deficiency anemia (IDA) has been recognized as the most common forms of malnutrition in developing countries^5^.

Child undernutrition (mainly involving stunting, wasting and underweight) is a serious global public health problem in developing world including Ethiopia^6^. Although, the magnitude of childhood undernutrition has decreased from 58% in 2000 to 40% in 2014 in Ethiopia, it continues as the major public health concern causing low academic performance in primary schools^2,7^.

In the study area, the implementation of mass deworming in line with vaccination for children began in 1998, and efforts to reach national coverage have intensified over the years (clinical records of the town, 2017). Despite these efforts, the prevalence of STH infections remains high in the town according to the clinical data generated from the health offices of the town. In the area, infection by *A. lumbricoides,* hookworm, and *T. trichiura* are the common STHs among school aged children. However, there is still a scarcity of adequate information on the prevalence and association of STH with undernutrition. Therefore, the present study attempted to determine the prevalence of STH and its association with undernutrition among schoolchildren attending Mettu town primary schools, Southwest Ethiopia.

## Materials and methods

### Study design

This studyutilized a cross-sectional study design.

### Study setting and study area

Mettu is a capital town for the district and Ilu Aba Bore zone. The town lies between 8°18′ N 35°35′ E and an altitude of 1605 m above sea level. The weather condition of the study area was characterized by an average temperature of 17 °C and 92% Humidity. According to the 2013 population projection report, the number of inhabitants was estimated to be 54,792 (22,857 males and 31,935 females). Agriculture, government employee, and small-scale trading are means by which the local people earn their living costs. In the town, eight primary schools are found and an estimated 5,000 school children were attending their education.

### Study population

All school children attending primary schools of Mettu town in the academic year 2018 were the source population. Children aged 5–16 years old, who were not terminally ill, having no any other disease conditions, not on anti-helminthic chemotherapy for the past 3 months and with no iron supplementation were included in the study.

### Sample size and sampling technique

The sample size for the study was determined using single population proportion formula; $n = {{z^{2} p\left ( {1 - p} \right)} \mathord{\left/ {\vphantom {{z^{2} p\left ( {1 - p} \right)} {d^{2} }}} \right. \kern-\nulldelimiterspace} {d^{2} }}$ at 95% confidence of interval (CI) where, *z* = 1.96, p = 0.5 and d = 5% precision (0.05). The prevalence of STH and undernutrition was not known among school age children in the study area. Thus, to calculate sample size of the study participants, 50% prevalence of STH and undernutritionwas assumed and the sample size estimated to be 384 children. Five percent of the total estimated sample size for withdraws or missed data were added and the final sample size was 404.Four schools equivalently admitted near to 50% of the total school children attended the primary education in the town was selected purposively from the list of schools and systematic random sampling technique was employed to select the sampling unit by using class attendance sheet. Finally, to ensure that data include independent observations, only a child was selected from a family.

### Nutritional status determination

Duplicate body weight and height data were taken by trained data collectors to evaluate the nutritional status of study participants. Height and weight measurements were recorded to the nearest to 0.1 cm and 0.1 kg respectively. School children wore light clothes and were bare footed during measurements. Weight for age (WAZ), height for age (HAZ), and body mass index (BMI) for age were compared with WHO child growth reference standards. The body mass index (BMI) for age is the index of choice for determining nutritional status for children above 5 years^8^. Study participants with Z-score values of < − 2SD for height for age and weight for height were classified as stunted and wasted respectively. Likewise, children with a Z-score value of < − 2 SD for weight for age were also classified as underweight.

### Parasitological examination

Stool samples were collected from study participants at the health post of the schools using labeled, sterile screw-capped plastic containers and transported to Mettu University parasitology laboratory within half an hour. Standard Kato-Katz technique (thick smear 41.7 mg) was employed to examine stool specimens for the presence of *A. lumbricoides*, *T. trichiura* and hookworm eggs. Microscopic examination was done within 1 h of Kato smear preparation for hookworms and after 2 h for *A. lumbricoides* and *T. trichiura*. The mean intensity of infection was expressed as the number of eggs per gram stool (EPG) and classified according to World Health Organization (WHO) criteria^9^. Double Kato-Katz slides were prepared from each stool specimen and examined twice by two different laboratory experts.

### Socio-demographic assessments

Semi-structured questionnaire and physical observation were used to collect information related with socio-demographic characteristics of study participants and associated factors.

### Data analysis

Data were double entered, cleaned in Microsoft Office 2010 and analysis done using Stata 11 statistical software (Stata Corporation, College Station, Texas, USA). The Chi-square test was employed to see the association of socio-demographic variable with the prevalence of STH infection. Logistic regression analysis was also conducted to assess the risk factors for nutritional status indices. At first, the effect of each exposure variable on the outcome was evaluated using bivariate logistic regression analysis. Then, variables with a significant association at 0.2 and less level of significance were considered for the final multivariate analysis using a backward stepwise model. Z-score for undernutrition were calculated using WHO 2007 AnthroPlus software^10^. P < 0.05 was considered statistically significant.

### Ethics approval and consent to participate

The study was carried out after having an ethical clearance endorsement from Mettu University Research Technical and Ethical Review Committee and all methods were carried out in accordance with relevant guidelines and regulations. In addition, informed written consent was obtained from guardian of study participants. Positive children for STH and other intestinal parasitosis got free treatment using standard dose of the respective drugs prescribed by a physician.

## Results

### Socio-demographic characteristics

Four hundred four schoolchildren aged from 5 to 16 years old were recruited for this study at the beginning. However, 12 (3%) children were excluded from the study because of missing stool specimens and labeling errors. The mean average age of study participants was 9.5 years (SD: ± 2.4 years) and 211 (53.8%) were female. Maternal literacy was found to be 37.2% and 45.2% for fathers. Families of the majority of study participants 289 (73.7%) had a latrine and 183 (46.7%) of the study participants were using tap water. One hundred twenty eight (32.6%) children were identified for undernutrition (Table 1).

**Table 1:** Socio-demographic characteristics of schoolchildren in Mettu town, Southwest Ethiopia, 2018 (n = 392).

Variables	Category	Frequency (n)	Percentage (%)
Age	5–10	192	48.9
	11–16	200	51.1
Sex	Male	181	46.2
	Female	211	53.8
Maternal literacy	Literate	146	37.2
	Illiterate	246	62.8
Paternal literacy	Literate	177	45.2
	Illiterate	215	54.8
Maternal occupation	Employed	93	23.7
	Unemployed	299	76.3
Paternal occupation	Employed	104	26.5
	Unemployed	188	73.5
Dirty fingernail	Yes	311	79.3
	No	81	20.7
Open filed defecation	Yes	289	73.7
	No	103	26.3
Undernutrition	Yes	128	32.6
	No	264	67.4
Types of undernutrition	Stunting	63	16.1
	Wasting	20	5.1
	Underweight	45	11.4

### STH infection among school-aged children

A total of 331 (84.4%) study participants were found positive for one or more STH infections. The most prevalent parasite identified was *A. lumbricoides* (39.0%) followed by *T. trichiura* (32.9%) and hookworm (28.1%). *S. mansoni* (2.3%), *H. nana* (1.8%), *Taenia* species (1.3%), and *E. vermicularis* (0.7%) infections were also detected among the study participants. According to the data presented in Table 2, a statistically significant (p < 0.05) association between STH with age groups, maternal literacy, fingernail status, and maternal education was observed. However, gender and habit of shoe wearing, paternal education and occupation, source of drinking water, and availability of latrine did not have any significant association with the presence of STH infections among study children (Table 2).

**Table 2:** Factors associated with soil transmitted helminthes infections among school children in Mettu town, Southwest Ethiopia, 2018 (n = 392).

Variables	Categories	STH infection status n (%)		
		*A. lumbricoides*	*T. trichiura*	Hookworm
Age	5–10	83 (36.6)	67 (29.5)	39 (17.2)
	11–16	46 (27.9)	42 (25.5)	54 (32.7)
	*X*^2^ (*P*)	5.91 (0.02)*	2.64 (0.06)*	0.82 (0.36)
Sex	Male	52 (28.7)	48 (26.5)	39 (21.5)
	Female	77 (36.5)	61 (28.9)	54 (25.6)
	*X*^2^ (*P*)	2.72 (0.09)	0.83 (0.35)	4.85 (0.12)
Maternal literacy	Literate	37 (25.3)	28 (19.2)	27 (18.5)
	Illiterate	92 (37.4)	81 (32.9)	66 (26.8)
	*X*^2^ (*P*)	9.21 (0.01)*	7.54 (0.00)**	17.6 (0.00)**
Paternal literacy	Literate	53 (29.9)	44 (24.8)	37 (20.9)
	Illiterate	76 (35.3)	65 (30.2)	56 (26.1)
	*X*^2^ (*P*)	2.23 (0.14)	0.12 (0.07)	3.63 (0.06)
Maternal occupation	Employed	43 (46.23)	34 (36.5)	24 (38.1)
	Unemployed	86 (28.7)	75 (25.1)	69 (23.1)
	*X*^2^ (*P*)	2.46 (0.12)*	1.63 (0.21)	0.13 (0.79)
Paternal occupation	Employed	54 (28.7)	48 (25.5)	50 (26.6)
	Unemployed	75 (36.7)	61 (29.9)	43 (21.1)
	*X*^2^ (*P*)	0.22 (0.71)	0.51 (0.45)	0.23 (0.98)
Dirty fingernail	Yes	103 (33.1)	88 (28.3)	68 (21.9)
	No	26 (32.1)	21 (25.9)	25 (30.9)
	*X*^2^ (*P*)	9.26 (0.001)*	4.82 (0.03)*	3.31 (0.04)*
Shoe wearing	Yes	101 (32.6)	82 (26.4)	65 ( 20.9)
	No	28 (34.1)	27 (32.9)	28 (34.1)
	*X*^2^ (*P*)	0.41 ( 0.52)	0.32 (0.59)	1.21 (0.28)
Water source	Tap water	55 (30.1)	53 (28.9)	46 (25.1)
	Others	74 (35.4)	56 (26.8)	47 (22.5)
	*X*^2^ (*P*)	0.32 (0.55)	0.12 (0.91)	1.61 (0.22)
Latrine availability	Yes	93 (32.2)	76 (26.3)	59 (20.4)
	No	36 (34.9)	33 (32.1)	34 (33.1)
	*X*^2^ (*P*)	7.14 (0.08)	3.72 (0.09 )	0.91 (0.41)

**Statistically significant at P < 0.01.

*Statistically significant at P < 0.05.

### Factors associated with STH infection and under nutrition

Bivariate logistic regression analysis showed that infection of STH was at higher risk for undernutrition than non-infected. *T. trichiura* infection was associated with significant increased risk of undernutrition (AOR 0.49, 95% CI 0.31, 0.83). Whereas, infection of *A. lumbricoides* and hookworm didn’t show any significant (P > 0.05) association with undernutrition although a higher prevalence of undernutrition was also seen among children being infected. Undernutrition was also found significantly associated with age (AOR 2.18, 95% CI 1.53, 6.59), maternal literacy (AOR 0.13, 95% CI 0.91, 0.34), and maternal occupation (AOR 1.67, 95% CI 1.08, 5.91). Children in the ages between 5 and 10 years were also about 3 times (95% CI 1.53, 6.59) more likely to be undernourished than children in the ages 11 to 16 years. Controlling for other variables, the final model retained having an illiterate mother (AOR 0.13, 95% CI 0.04, 0.35) (p < 0.001) as the major factor associated with undernutrition (Table 3).

**Table 3:** Results of bivariate and multivariate Logistic regression analysis for undernutrition among school children in Mettu town, Southwest Ethiopia, 2018 (n = 392).

Variable	Nutritional status (n)		COR (95% CI)	AOR (95% CI)
	Undernutrition	Normal		
**Age**				
5–10	78	114	3.21 (1.53–6.59)**	2.18 (1.02–4.83)*
11–16 (ref.)	50	150	1	
**Sex**				
Male	76	105	1.09 (00.82–2.81)	0.88 (0.16–1.91)
Female (ref.)	52	159	1	
**Maternal literacy**				
Literate	38	108	0.26 (0.91–0.34)**	0.13 (0.04–0.35)**
Illiterate (ref.)	90	156	1	
**Paternal literacy**				
Literate	55	122	2.14 (1.01–3.72)	1.27 (0.93–2.48)
Illiterate (ref.)	73	142	1	
**Maternal occupation**				
Employed	19	74	2.53 (1.08–5.91)**	1.67 (0.81–3.05)
Unemployed (ref.)	109	190	1	
**Paternal occupation**				
Employed	58	130	1.04 (0.72–2.68)	0.78 (0.38–1.61)
Unemployed (ref.)	70	134	1	
**Dirty finger nail**				
Yes	99	212	2.53 (1.18–4.13)	1.12 (0.06–2.17)
No (ref.)	29	52	1	
**Drinking water sources**				
Tap water (ref.)	71	112	1	2.18 (1.22–3.16)
Others (well, spring, river)	57	152	2.07 (1.45–2.71)	
**Shoe wearing habit**				
Yes	97	213	1.36 (0.97–1.62)	1.15 (1.02–1.99)
No (ref.)	31	51	1	
**Availability of latrine**				
Yes	94	195	1.25 (1.01–2.03)	1.44 (1.07–2.09)
No (ref.)	34	69	1	
***A. lumbricoides***				
Yes	68	61	1.09 (0.87–2.91)	0.87 (0.42–1.79)
No (ref.)	60	203	1	
***T. trichiura***				
Yes	70	39	0.49 (0.31–0.83)**	0.52 (0.30–0.91)*
No (ref.)	58	225	1	
**Hookworm**				
Yes	54	39	2.01 (1.37–2.67)	2.06 (1.18–3.09)
No (ref.)	74	225	1	

*COR* crude odd ratio, *AOR* adjusted odd ratio.

**Statistically significant P < 0.01.

*Statistically significant P < 0.05.

## Discussion

STH infections and malnutrition are among the most common public health problems, primarily affecting children living in rural and semi-urban areas of developing countries^1,2^. Given this, the present study attempted to determine the prevalence of STH infections and undernutrition among schoolchildren in Mettu town, Southwest Ethiopia. The results then revealed the high prevalence of STH infections (84.4%) which is higher than the findings from “Butajira”^11^ and “Jimma”, Ethiopia^12^. However, the result of this study was comparable with the reports from Nigeria (83.3%)^13^ and the Philippines (82.8%)^14^. In contrast, it was much higher than the study conducted in the west-central border area of Thailand (15.6%)^15^. These differences could be explained by variations in altitude, hygienic conditions, awareness level towards STH and playing habits and facilities of children in and outside the school^11^.

The socioeconomic status of the parents did not measured directly as many of them could not give accurate information about their income. However, the occupation and educational background of the parents and physical observation of the fingernail status of children enrolled could help as an indicator of their socioeconomic status^11^. As a result, *A. lumbricoides* infection was found significantly associated with age groups, maternal literacy, and fingernail status, while*, T. trichiura* and Hookworm infection was associated with maternal literacy, maternal occupation and fingernail status of children. This reflects the low socioeconomic status and unhygienic living conditions which lead to STH infectious agents flourished in the study area. The other point is paternal literacy status does not show any association with STH infections and the justification could be implicated to the different in roles fathers and mothers play in the management of the family and mothers play a crucial role on hygiene and feeding activities.

The prevalence of *A. lumbricoides* (39.0%), *T. trichiura* (32.9%), and Hookworm (28.1% %) shown in this study were very much higher than the national prevalence of STH infection report and the findings of other similar studies^11,12,16^. *A. lumbricoides* infections were found to be significantly higher in children of ages 5 to 10 years than children of ages 11 to 16 years (P = 0.02, x^2^ = 5.91). It shows children in the age groups of 5–10 years are more exposed to the infection. This could be justified as children below the age of 10 years playing in the contaminated outdoor environment and experience frequent contact with soil. It is because when age increases, the prevalence of STH infection decreases possibly due to improved personal hygiene and reduced contact with soil. Reports from Nigeria^13,17^ also showed the highest prevalence of infections among the younger age groups. However, in contrary to this result, *A. lumbricoides* infection was found to be higher among children aged > 10 years reported from the Philippines^14^. The other justification for the higher prevalence of *A. lumbricoides* in the study area is could possibly be the humid climatic condition and clay nature of the soil which is favorable for the ova to survive longer^18^.

In the present findings, the prevalence of stunting, underweight, and wasting was found to be (16.1%), (5.1%), and (11.4%) respectively among the study participants. It was considerably lower than the report of the Ethiopian Demographic Health Survey (EDHS) which was (51.3%), (35.7%), and (9.7%) respectively^9^. A similar study from Mongolia^19^ showed a lower rate of undernutrition (29.3%), while a report from Nigeria; similar figures with the present finding were determined^17^. These different findings could be attributed to the difference in agro-ecology, socioeconomic status of the study participants, and the type of staple food the community depends on (vegetable, fruits and roots of some plant species which are rich in fiber proteins and vitamins).

Infection of *T. trichiura* was found significantly associated with the prevalence of undernutrition. Medium and heavy intensity infections of *T. trichiura* have contributed to its association with undernutrition because adult worm of the parasite is known to play a significant role through gastrointestinal blood loss and immunological disturbances^20^. Thus, the finding is consistent with the work of Shang et al., who reported that moderate to heavy intensity of *T. trichiura* infection identified as factors for the occurrence of high stunting among school children in China^21^.

The prevalence of undernutrition was significantly higher among children aged 5–10 years compared to those aged 11–16 years. A similar previous study also documented the same result with the present study^14^. The high nutritional requirement associated with rapid growth and developments in young children affects their nutritional status and normal growth. In this study, children born from mothers who didn’t have formal education were more likely to be undernourished than their counterparts (AOR 0.13, 95% CI 0.04, 0.35) (p = 0.015). This was in line with the findings from India^22^ and Bolivia^23^ which revealed that uneducated mothers were likely to have malnourished children than those of the educated. This could be expected that educated mothers might have good knowledge on health, hygiene, and feeding practice of their children than uneducated.

## Conclusion

The result of the present study showed the high prevalence of STH and undernutrition. Risk of STH was increased with maternal illiteracy, maternal unemployment, under ten years of age and dirty fingernail of the study participants. Undernutrition was also found significantly associated with STH. The study warrant the need of integrated control programs including mass deworming campaign, synchronized nutritional rehabilitation, and maternal health education to curve the problem.

## Limitation of the study

The present study used only anthropomorphic measurements to collect nutritional status and did not assess the micronutrient parameters of study participants. Hence, this finding should be interpreted by taking into account these limitations.

## Data Availability

The datasets used during the current study are available from the corresponding author on reasonable request.

## Abbreviations

AOR: Adjusted odds ratio
BAZ: Body-mass-index-for-age
BMI: Body mass index
CI: Confidence interval
DHS: Demographic and Health Survey
EPG: Eggs per gram of faeces
HAZ: Height-for-age
Hb: Haemoglobin
HAZ: Height for age
IDA: Iron deficiency anemia
PEM: Protein-energy malnutrition
SD: Standard deviation
STH: Soil-transmitted helminths
WAZ: Weight-for-age

## References

[1] Stephenson LS, Latham MC, Ottesen EA (2000). Malnutrition and parasitic helminth infections. Parasitology.

[2] Hotez PJ, Brindley PJ, Bethony JM, King CH, Pearce EJ, Jacobson J (2008). Helminth infections: The great neglected tropical diseases. J. Clin. Invest..

[3] Ezeamama AE, Friedman JF, Acosta LP, Bellinger DC, Langdon GC, Manalo DL, Olveda RM, Kurtis JD, McGarvey ST (2005). Helminth infection and cognitive impairment among Filipino children. Am. J. Trop. Med. Hyg..

[4] Sorensen WC, Cappello M, Bell D, Difedele LM, Brown MA (2011). Polyhelminth infection in East Guatemalan school children. J. Glob. Infect. Dis..

[5] Stephenson LS, Latham MC, Ottesen EA (2000). Global malnutrition. Parasitology.

[6] Tariku A, Woldie H, Fekadu A, Adane A, Ferede A, Yitayew S (2016). Nearly half of preschool children are stunted in Dembia district, Northwest Ethiopia: A community based cross sectional study. Arch. Public Health.

[7] Central Statistical Agency and ICF. *Ethiopia Demographic and Health Survey 2016: Key Indicators Report* (CSA and ICF, 2016). https://dhsprogram.com/pubs/pdf/FR328/FR328.pdf.

[8] WHO (1997). Global Database on Child Growth and Malnutrition.

[9] Montresor A, Crompton DWT, Hall H, Bundy DAP, Savioli L (1998). Guidelines for the Evaluation of Soil-Transmitted Helminthiasis and Schistosomiasis at a Community Level. A Guide for Control Program Managers, WHO/CTD/SIP981.

[10] WHO. *AnthroPlus Software*. https://www.who.int/tools/growth-reference-data-for-5to19-years/application-tools (2007).

[11] Belyhun Y, Medhin G, Amberbir A (2010). Prevalence and risk factors for soil-transmitted helminth infection in mothers and their infants in Butajira, Ethiopia: A population-based study. BMC Public Health.

[12] Debalke S, Worku A, Jahur N, Mekonnen Z (2013). Soil-transmitted helminths, and associated factors among schoolchildren in government and private primary school in Jimma town, southwest Ethiopia. Ethiop. J. Health Sci..

[13] Ibidapo CA, Okwa O (2008). The prevalence and intensity of soil transmitted helminths in a rural community, Lagos suburb, southwest Nigeria. Int. J. Agric. Biol..

[14] Papier K, Williams GM, Luceres-Catubig R, Ahmed F, Olveda RM, McManus DP, Chy D, Chau TN, Gray DJ, Ross AG (2014). Childhood malnutrition and parasitic helminth interactions. Clin. Infect. Dis..

[15] Anantaphruti MT, Waikagul J, Maipanich W, Nuamtanong S, Pubampen S (2004). Soil-transmitted helminthiases and health behaviors among schoolchildren and community members in a west-central border area of Thailand. Southwest Asian J. Trop. Med. Public Health.

[16] Mathewos B, Alemu A, Woldeyohannes D, Alemu A, Addis Z, Tiruneh M, Aimero M, Kassu A (2014). Current status of soil transmitted helminths and *Schistosomamansoni* infection among children in two primary schools in North Gondar, Northwest Ethiopia: A cross-sectional study. BMC Res. Notes.

[17] Senbanjo IO, Oshikoya KA, Odusanya OO, Njokanma OF (2011). Prevalence of and risk factors for stunting among school children and adolescents in Abeokuta, southwest Nigeria. J. Health Popul. Nutr..

[18] Yeshanew S, Tadege M, Abamecha A (2021). Prevalence and associated factors of intestinal parasitic infections among food handlers in Mettu Town, Southwest Ethiopia. J. Trop. Med..

[19] Otgonjargal D, Bradley A, Woodruff F, Batjarga IDD (2012). Nutritional status of under-five children in Mongolia. J. Med. Med. Sci..

[20] Stephenson LS, Holland CV, Cooper ES (2000). The public health significance of *Trichuris trichuira*. Parasitology.

[21] Shang Y, Tang LH, Zhou SS, Chen YD, Yang YC, Lin SX (2010). Stunting and soil-transmitted-helminth infections among school-age pupils in rural areas of southern China. Parasites Vectors.

[22] Helga BU, Torill B, Maurice BM (2011). Socioeconomic status and chronic child malnutrition: Wealth and maternal education matter more in the peruvian andes than nationally. Nutr. Res..

[23] Frost MB, Forste R, Haas DW (2005). Maternal education and child nutritional status in Bolivia: Finding the links. Soc. Sci. Med..

